# The Air Stability of Sodium Layered Oxide NaTMO_2_ (100) Surface Investigated via DFT Calculations

**DOI:** 10.3390/nano15141067

**Published:** 2025-07-10

**Authors:** Hui Li, Qing Xue, Shengyi Li, Xuechun Wang, Yijie Hou, Chang Sun, Cun Wang, Guozheng Sheng, Peng Sheng, Huitao Bai, Li Xu, Yumin Qian

**Affiliations:** 1 Beijing Institute of Smart Energy, Beijing 102209, Chinalishengyi@bise.hrl.ac.cn (S.L.); baihuitao@bise.hrl.ac.cn (H.B.); 2 Key Laboratory of Advanced Optoelectronic Quantum Architecture and Measurement, Ministry of Education, School of Physics, Beijing Institute of Technology, Beijing 100081, China

**Keywords:** DFT, surface electron structure, air stability, sodium ion battery

## Abstract

Air stability caused by the H_2_O/CO_2_ reaction at the layered oxide NaTMO_2_ surface is one of the main obstacles to commercializing sodium-ion batteries (SIBS). The H_2_O and CO_2_ adsorption properties on the (100) surface of sodium layered transition metal oxide NaTMO_2_ (TM = Co, Ni, Mo, Nd) are calculated using the DFT method to study the surface air stability. This study showed that the material bulk phase (symmetry), surface site, element type, and surface termination are all (though not the only) important factors that affect the adsorption strength. Contrary to previous studies, the P phase is not always more air-stable than the O phase; our calculations showed that the NaNiO_2_ O phase is more stable than the P phase. The calculated band center and occupation showed a direct relationship with the adsorption energy. The Na site adsorption for CO_2_ and H_2_O showed the same V-shape trend. However, the TM adsorption for CO_2_ and H_2_O showed a different trend. With an increased t_2_g band center, CO_2_ adsorption strength increases. There is no clear trend for H_2_O adsorption. Our calculations showed that the electronic structure of the surface atomic of adsorption site plays a decisive role in CO_2_ and H_2_O adsorption strength. This study demonstrated an effective method for obtaining a stability parameter regarding the electronic structure, which can be used to screen the air-stable layered oxide sodium cathode in the future.

## 1. Introduction

Fossil fuels, such as coal, oil, and natural gas, lay a critical material foundation for human survival and development, supporting societal progress and improving living standards. However, the excessive demand for fossil energy generates environmental pollution and global warming issues. Thus, searching for green and renewable alternatives (solar, wind, tidal energy, etc.) is a priority towards building a sustainable future [1,2,3]. Battery energy storage is preferred over other energy storage systems (ESSs) due to its high efficiency, long cycle life, and easier transmission via smart grids with high energy conversion efficiency [4]. With technological advances, the lithium-ion battery (LIBS) market has greatly expanded over the past ten years with the emergence of various electronic mobile devices, vehicles and the widespread applicatedenergy storage devices [5,6]. Nevertheless, continuously supplying LIBS is challenging due to the inadequate and maldistributed lithium in the Earth. In contrast, sodium has physical and electrochemical properties similar to lithium, but it is more abundant, with a crustal abundance of 2.64%. Therefore, sodium-ion batteries (SIBS) are gradually gaining more attention as a rechargeable battery alternative to lithium due to their advantages, including low cost, high safety, and high energy density.

A global effort has been made to accelerate SIBS commercialization. The cathode material is the well-known key factor that determines SIBS energy density [7,8], which can be primarily divided into polyanionic compounds, layered transition metal oxides, Prussian blue analogs, and metal–organic materials [9]. Among them, layered transition metal oxides have generally been used by virtue of their simple structure, facile synthesis, and high specific capacity. However, some challenges limit further adoption of layered transition metal oxides, such as air instability, short lifespan, and phase transition during battery cycling [9,10,11]. In particular, high air sensitivity has gained significant attention since it is the intrinsic spark that causes many negative effects, such as structure instability, capacity decay, and cycling reduction [12,13]. Thus, we must study the origin of air instability and take measures to prevent these negative effects [12]. To accelerate the commercialization of sodium-ion batteries, it is particularly important to explore the air stability of layered oxide cathode materials. Pan’s group comprehensively studied O3-phase NaNi_1/3_Fe_1/3_Mn_1/3_O_2_ materials under atmospheric conditions by controlling humidity and temperature and found that carbon dioxide was initially inserted into the sodium layer along the material surface (003), causing sodium carbonate to begin to grow between the metal layers, which further led to material structure destruction [14]. Pan et al. also found that the edges of (003) were more susceptible to moist air than the planes of (003) [14]. Hu’s group synthesized the O3-phase Na_0.9_[Cu_0.22_Fe_0.30_Mn_0.48_]O_2_ material without traditional Co and Ni and found that it was very stable in aqueous environments [12]. The reason for this is that both copper and iron are electrochemically active, and Cu^2+^/Cu^3+^ and Fe^3+^/Fe^4+^ redox is mainly responsible for the charge compensation mechanism, introducing copper into the layered oxide, increasing the average storage voltage, to avoid oxidation, along with the formation of different surface structures and compositions, thus protecting the material from direct contact with air. Malachi et al. studied the high-entropy and Co-free sodium-ion layered fluoride oxide and found that introducing Li to replace part of Na can reduce the shielding effect of sodium ions, thereby enhancing the oxidation resistance of the material and inhibiting the harmful reaction between the material and air. Among them, Na_0.9_Li_0.1_ is particularly prominent [15]. Huang et al. reported that Li/Mg co-doped Manganese/nickel-based layered transition metal oxides showed enhanced cycling stability due to the O-2p nonbonding states from Li and Mg–O bonds stabilizing the Ni–O eg states [16]. Manthiram et al. found that adding a 1 wt % (more than 1% reduces initial capacity) (NaPO_3_)n coating to O3-phase (Ni_0.3_Fe_0.4_Mn_0.3_)O_2_ materials can significantly improve their air stability, as sodium polyphosphate is converted into water and sodium phosphate into moist air to protect the layered structure [17]. Wei et al. also reported that O_3_-type Na_0.96_Ca_0.02_Ni_0.25_Fe_0.5_Mn_0.25_O_2_ delivered a reversible capacity of 122.1 mAh·g^−1^ at 10 mA·g^−1^, with a capacity retention of 83.4% after 200 cycles at 50 mA·g^−1^ and a good rate capability [18]. Zhang et al. also reported that a Ca-doped O_3_-type phase material with different ratios of Ni/Fe/Mn showed good air stability [19].

At the same time, researchers have been studying the air stability of P-phase layered materials. Chen et al. reported that Nb-doped Na_0.67_Mn_0.67_Ni_0.33_Nb_0.03_O_2_ could exhibit superior performance and outstanding cycling in a half cell after exposure in a moisture atmosphere (RH 93%) for 20 days [20]. Yin et al. observed that a Li-doped P2-type Na_0.67_Li_0.1_(Mn_0.7_Ni_0.2_Cu_0.1_)_0.9_O_2_ (NLMNC) cathode demonstrated good cycling performance after storage in air for 7 days, almost overlaying that of the pristine sample [21]. Liu et al. reported an air-stable single-crystal P2-type Na_2/3_Ni_1/3_Mn_1/3_Ti_1/3_O_2_ with reversible phase transitions (P2-OP4) [22]. Yang et al. used a simple second-sintering strategy to enhance NFM air stability by removing impurities and retrieving surface-precipitated Na^+^from the crystalline host [23]. Gu et al. designed and synthesized a novel air-stabilized P2-phase Na_7/9_Cu_2/9_Fe_1/9_Mn_2/3_O_2_, which has excellent reversible capacity (89 m Ah g^−1^ at 0.1c) and cycling stability (85% capacity retention after 150 cycles at 1c) [24]. Chen et al. compared P2-phase Na_0.6_MnO_2_ and Na_0.6_Mn_0.9_Cu_0.1_O_2_ and found that doping Cu^2+^ can effectively inhibit the Jahn–Teller effect of Mn^3+^ and improve the Na^+^/vacancy-ordering transition, thereby enhancing its air stability [25]. Zhou et al. designed and successfully prepared P3-phase Na_2/3_Ni_1/4_Mg_1/12_Mn_2/3_O_2_ sodium-ion battery cathode material, which is a good high-voltage air-stabilized material with high air stability (especially able to resist a large amount of water) and electrochemical utilization, and it can inhibit the phase transition at high voltage, thus realizing high-efficiency sodium storage [26]. Zuo et al. successfully extended the Na^+^ layer spacing of P2-phase Na_0.67_MnO_2_ via a water-mediated method (which is also feasible in other sodium-ion layered oxides), improving structural stability and Na^+^ mobility [27]. The material formed a hydrated phase with water in humid air and thus has good moisture resistance for atmospheric storage [27].

An increasing number of materials are being reported with different degrees of air stability. However, we are still lacking a structural parameter that measures stability. The reason for the poor air stability (mainly water and carbon dioxide) of sodium layered oxide materials is also not clear. In this study, we investigated the air stability of 3d- NaCoO_2_ and NaNiO_2_, 4d- NaNbO_2_ [28,29] and NaMoO_2_ [30,31] materials through DFT calculations for their O and P phases [32,33,34], respectively, which include a series of calculations of surface energies, work functions of the surface and adsorption energies of water and carbon dioxide [35]. More importantly, the O3 phase provides additional sodium storage sites, thereby increasing the material’s capacity over the P2 phase. However, previous studies showed that the O3 phase encounters a more severe air stability problem than the P2 phase. Thus, the exploration method for establishing an air stabilization strategy for the O3 phase is also very important. Our DFT calculation study contradicted the general belief that the P2 phase is always more stable than the O3 phase. Symmetry is not the only factor that determines air stability. This study outlines the key roles of the eletron structure for the determination of adsorption energy of the CO_2_/H_2_O, which will open a new door to searching and tuning for new stable O3-phase layered materials with higher capacities.

## 2. Computation Details

First-principle calculations, based on the density functional theory (DFT), were performed with generalized gradient approximation (GGA). Core electron states were represented by the projector augmented-wave method [36], as implemented in the Vienna ab initio simulation package (VASP) [37]. The R^2^SCAN [38] exchange correlation functional and a plane-wave representation for the wave function with a cut-off energy of 450 eV were used. The experimental lattice parameter was adopted, and the atomic position was fully relaxed and optimized with an atomic force convergence of 0.01 eV/Å and energy convergence of 0.1 meV before electron structure and total energy calculations.

The surface energy of a flat plate model with the Miller exponent (hkl) is given as follows [39]:

$$\gamma =\frac{E_{slab}-E_{bulk}\times N-E_{\left(Na/TM/O\right)}\times N_{\left(Na/TM/O\right)}}{2\times A_{slab}}$$

where E_slab_ is the total energy of the plate model, E_bulk_ is the energy of unit cell, E_(Na/TM/O)_ is the chemical potential of the excess atoms contained in the slab, N is the number of cells in the plate model, and A_slab_ is the plate surface area.

H_2_O and CO_2_ adsorption energy calculation is expressed as follows:

$$E=E_{*+mol}-E_{*}-E_{mol}$$

where $E_{*}$ is the surface energy, and $E_{mol}$ is the H_2_O or CO_2_ energy.

## 3. CO_2_ and H_2_O Adsorption Energy

It is generally accepted that adsorption energy above and below −1 eV yields weak physisorption and stronger chemisorption, respectively [40]. According to the definition of associative and dissociative adsorption [41], dissociative adsorption will include bond dissociation energy. The bond dissociation energies of H_2_O and CO_2_ are all above −5 eV [42,43]. After conferring with our experimental experts, we think it is safe to set −2.0 eV as the threshold energy for the non-dissociative adsorption of H_2_O and CO_2_. The experimental temperature spectra suggest that molecules with a calculated adsorption energy of −2.5 eV (250 KJ/mol) at 500 K [44] will be thermally activated for desorption when temperatures rise above 500 K. For sodium layered oxides with H_2_O and CO_2_ adsorption, if non-dissociative adsorption occurs on the crystal surface, they can be easily removed by simply putting the material into the furnace. Thus, a H_2_O/CO_2_ adsorption energy stronger than −2.0 eV is treated as a destructive dissociative adsorption indicator, which causes severe structural degradation and air stability issues. On the other hand, H_2_O/CO_2_ adsorption energy above −2.0 eV is treated as a non-dissociative adsorption indicator that adsorbents can be easily removed via thermal activation. In the present calculation, when the H_2_O/CO_2_ adsorption energy is around 2.0 eV, and no bond dissociation is observed, the above hypothesis is applicable.

To give an atomic-scale perspective of the above calculated air stability, we chose the most widely reported structure of O and P phases to calculate CO_2_ and H_2_O adsorption on the surface [45]. NaCoO_2_, NaNiO_2_, NaMoO_2_, and NaNbO_2_ are selected for the 3d and 4d transiton metal (TM) layered oxides. Most previous studies have been dedicated to the (001) surface [46,47]. Here, we choose the (100) surface, from which CO_2_ and H_2_O are most easily intercalated into the layered structure and lead to structural deterioration. Such a process has a decisive role for the structure stability that is of interest to the community in Na battery research.

**Figure 1:**
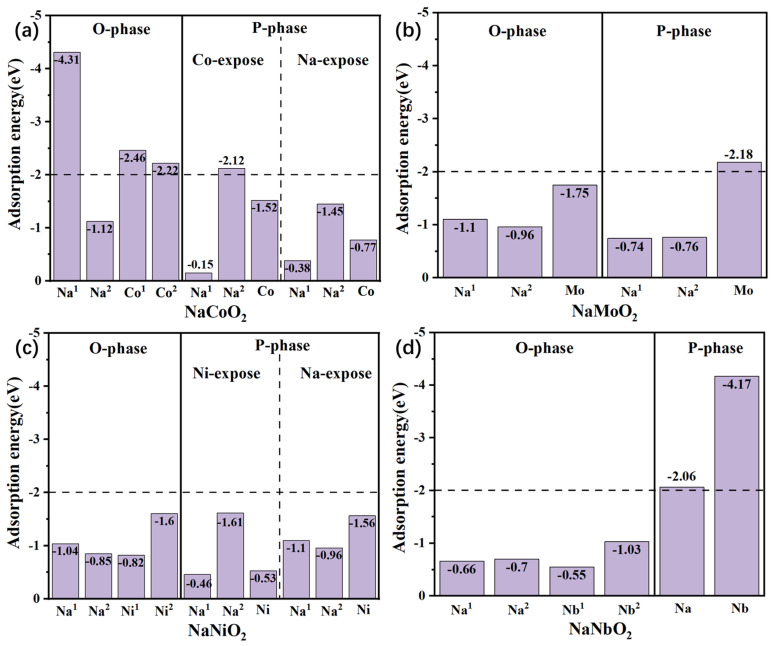
The CO_2_ adsorption energy of Na and TM atoms with different (100) surfaces.

The NaCoO_2_ (100) surface with an R $\bar{3}$m symmetry structure demonstrated very strong CO_2_ adsorption at the Na^1^ site, the calculated structure (Figure S4) showed CO_2_ triple-site adsorption, and the exposed Co atoms also showed strong adsorption for CO_2_. However, for the P63/mmc symmetry crystal, CO_2_ adsorption is much weaker, except for the Na^2^ site where the Na atom is coordinated by four oxygen atoms to form a quadrangular pyramid with the Na atom on top. From the CO_2_ molecule adsorption energy (Figure 1), it can be concluded that the P2-phase NaCoO_2_ is more stable than the O phase, which is consistent with past experiments [48,49]. The calculated CO_2_ adsorption energy on NaNiO_2_ shows a relatively small value for both the P and O phases. The CO_2_ adsorption on the reconstructed (100) surface showed a maximum of 1.61 eV. Such low energy indicates that NaNiO_2_ is passivated to CO_2_. For the 4d transition metal layered oxides, the O- and P-phase NaMoO_2_ showed maximum CO_2_ adsorption energies of −1.75 eV and −2.18 eV on the Mo site, respectively. The calculation showed that NaMoO_2_ has a more stable O phase than P phase in the CO_2_-rich atmosphere. Such a trend is more prominent in the NaNbO_2_ phase, where the P-phase (100) surface showed strong CO_2_ adsorption. However, CO_2_ molecule adsorption only showed very weak physisorption below −1.0 eV on the O-phase NaNbO_2_ surface.

**Figure 2:**
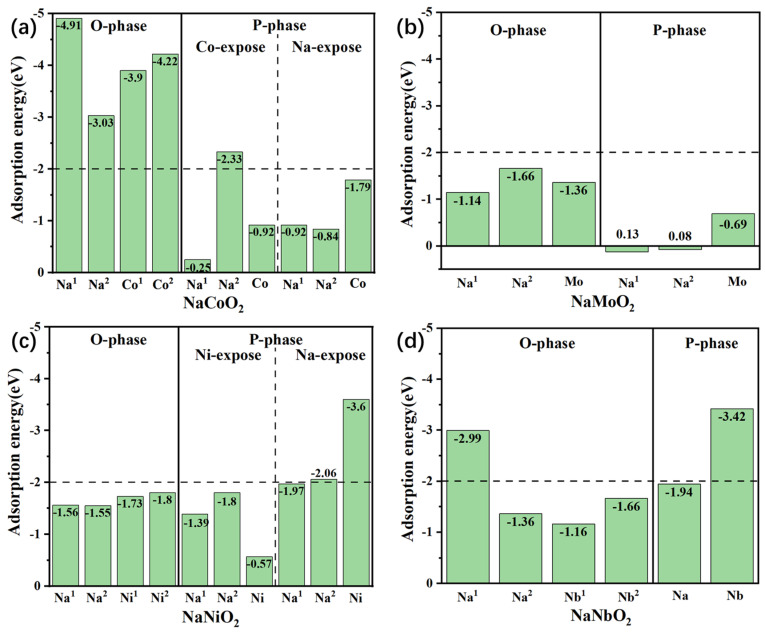
The H_2_O adsorption energy of Na and TM atoms with different (100) surfaces.

From the CO_2_ molecule adsorption energy (Figure 2), NaCoO_2_ with O-phase (100) surfaces showed very strong adsorption for the water molecule, along with OH formation after geometric optimization, as shown in Figure S4. However, for the P phase, the most strongly adsorbed site is the four-oxygen-coordinated Na site with an adsorption energy of −2.33 eV. Furthermore, most adsorption sites showed physisorption with adsorption energies below −1.0 eV. For the O-phase NaNiO_2_, water molecule adsorption is stronger but above −1.8 eV. On the other hand, P-phase adsorption showed much stronger H_2_O molecule binding to the Na site, especially from the Ni site (as shown in Figure S8). For NaMoO_2_, both the O and P phases showed relatively weak adsorption above −1.66 eV, especially for the P phase, in which water molecules only showed physisorption. NaNbO_2_ showed strong H_2_O adsorption for both the O and P phases, especially on the Na and Nb sites for the O and P phases, respectively.

The above calculations show that P-phase NaCoO_2_ is much more stable than the O phase, which is consistent with the general experimental results [49]. However, for NaNiO_2_, the calculation showed the opposite trend: the O phase is more stable than the P phase under open-air conditions. O-phase NaMoO_2_ shows good air stability, but the P phase shows less stability in the CO_2_-rich atmosphere. NaMoO_2_ is a very special material according to the present calculation, as it showed the most appealing adsorption energy for both CO_2_ and H_2_O, irrespective of phase structure and surface. Delmas did not report any air stability issues for the NaMoO_2_ cathode. Additionally, O-phase NaNbO_2_ showed strong stability in a CO_2_-rich atmosphere, but the P phase showed strong CO_2_ adsorption. Furthermore, neither the O nor P phase showed surface stability in the moist atmosphere.

## 4. Discussion

**Figure 3:**
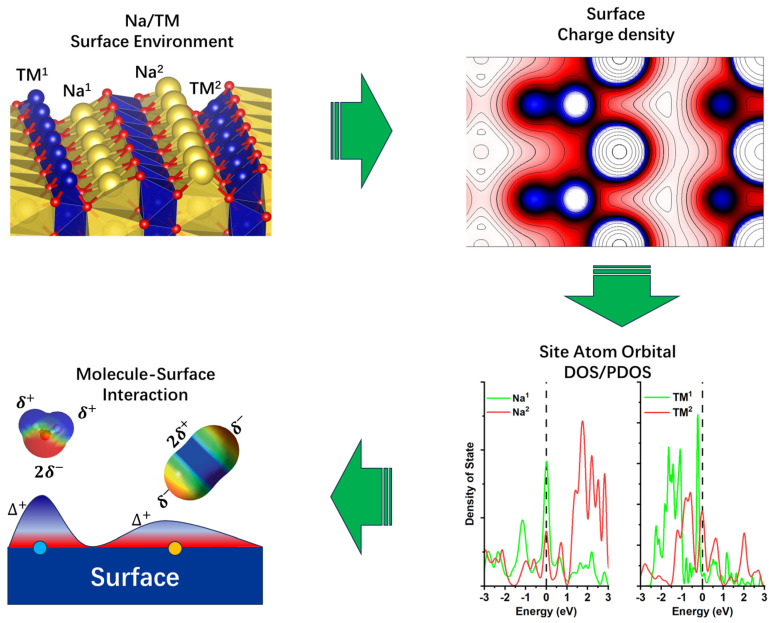
The illustration for the in-depth investigation of the H_2_O/CO_2_–surface interaction.

The molecule–solid interaction, also called the metal–support interaction [50,51] and d-band model [51], has been widely studied in the community invested in single-atom catalyst research. Many factors impact interaction strength, such as the surface charge density distribution, atom-site environment, surface-atom DOS, etc., as shown in Figure 3.

In the following section, a detailed analysis of the local atomic environment of the adsorption site and the charge distribution will be outlined. Along with the Na/TM surface adsorption site projected density of state (PDOS) is also given to aid the analysis of possible H_2_O/CO_2_ chemical bonding with the Na/TM adsorption site.

## 5. Surface Atomic Environment

**Figure 4:**
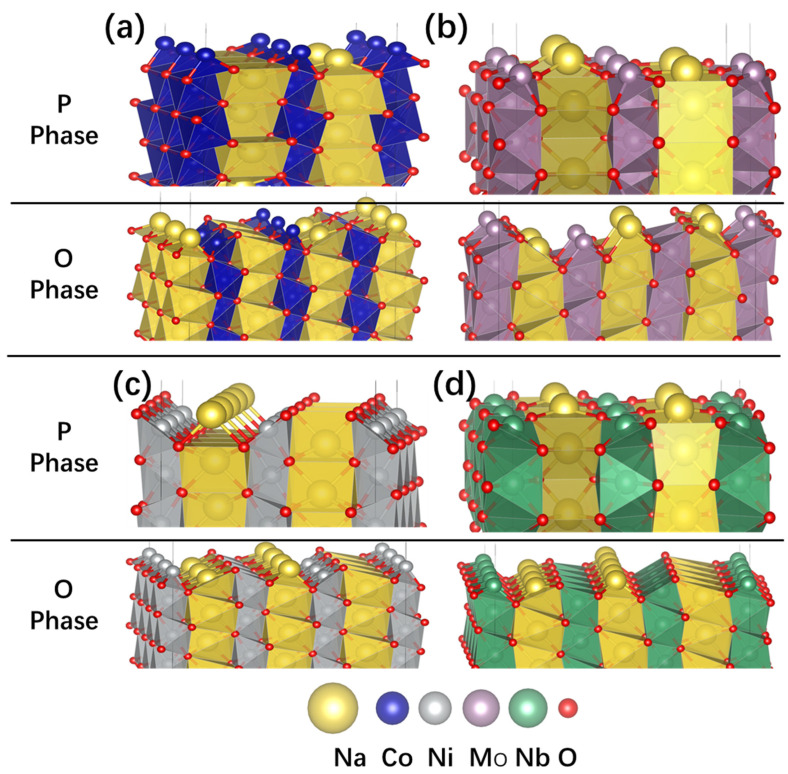
The (100) surface of NaTMO_2_: (**a**) NaCoO_2_, (**b**) NaNiO_2_, (**c**) NaMoO_2_, and (**d**) NaNbO_2_. The upper and lower panels are the P2 and O3 phases, respectively. This figure only shows the lower energy surface of P-phase NaCoO_2_ and NaNiO_2_ with Na/TM termination. The other higher atomic termination surfaces are given in Figure S2 in the Supporting Information.

The (100) surface of the selected material with P- and O-phase structures, downloaded from the material project [52], was cleaved using VESTA [53], as shown in Figure 4 and Figure S1. For the P-phase NaCoO_2_ and NaNiO_2_ (100) surface, there are two kinds of surfaces with different terminations: Na/TM termination (as shown in Figure 4) and Na/O termination (as shown in Figure S2). The calculated Na/Co termination has a surface energy of 0.1756 eV/Å^2^, which is lower than the corresponding Na/O termination with a surface energy of 0.322 eV/Å^2^.

However, for the O-phase NaCoO_2_ and NaNiO_2_ (100) surfaces, there is only one kind of surface with Na/TM/O termination, as shown in Figure 4a,b. For the P-phase NaCoO_2_, the Co-O atoms form a three-oxygen-coordinated triangular pyramid with Co at the top of the summit on the (100) surface. The Na atom is coordinated by four oxygen atoms with a non-planar structure. For the O-phase NaCoO_2_, the surface Co atoms have two kinds of local atomic environments on the (100) surface: a three-oxygen-coordinated triangular pyramid with Co at the top of the summit and a five-oxygen-coordinated square pyramid. The surface Na also has two kinds of local structures: a five-oxygen-coordinated square pyramid with Na at the center of the plane and a three-oxygen-coordinated triangular pyramid with Na at the top of the summit.

For the P-phase NaNiO_2_, the surface Ni-O atoms form a five-coordinated square pyramid, and Na is coordinated by two O atoms, as shown in Figure 4b. For the O-phase NaNiO_2_, the surface Ni-O atoms form a five-oxygen-coordinated square pyramid with Ni at the center of the plane and a three-oxygen-coordinated triangular pyramid with Ni at the top of the summit. The surface Na also has two kinds of local structures: a five-oxygen-coordinated square pyramid with Ni at the center of the plane and a three-oxygen-coordinated triangular pyramid with Ni at the top of the summit.

For the 4d Mo and Nb layered oxides, the P phase has cmcm (space group 63) symmetry, which differs from the 3d Na and Ni layered oxides. There is only one termination type, as shown in Figure 4c,d. The surface energy is 0.060 eV/Å^2^ for the P-phase NaMoO_2_ (100) surface. There are two kinds of Na sites: one coordinated with four oxygen atoms and the other with two oxygen atoms. The Mo stie has four O coordinated atoms with Mo atom at the center of the defective trigonal bipyramid. For the P-phase NaNbO_2_ with a surface energy of 0.061 eV/Å^2^, there is only one kind of Na site with four oxygen coordinates, and the Nb atom is located at the center of the defective trigonal bipyramid.

For the O-phase NaMoO_2_ and NaNbO_2_ with an R $\bar{3}$m symmetry, there is only one surface termination—the same as the O phase of Ni and Co layer oxides. The surface energies are 0.082 eV/Å^2^ and 0.105 eV/Å^2^, which are larger than their P-phase counterparts. For NaMoO_2_, there are two Na sites: one coordinated with four oxygen atoms and the other with three. The Mo atom is coordinated by three oxygen atoms in the Mo site. For NaNbO_2_, the Na atomic coordinate environment is the same as NaMoO_2_, but Nb has two sites, with one coordinated by three O atoms and the other by five.

From the analysis of the surface Na and TM atomic environment, the surface of 3d and 4d layered oxides showed various atomic environments. The difference in the Na/TM coordination environment indicates that the NaO_x_ and TMO_x_ polyhedron will change according to surface termination, symmetry, lattice parameters, and *d*-orbital shape. The crystal field split changes with NaO_x_ and TMO_x_ polyhedron changes; thus, the orbital overlap changes according to the crystal field. The previous study showed that surface MoC_x_ polyhedron changes will alter the surface H adsorption due to the Mo site electron structure change [54]. In the present case, the NaTMO_2_ surface showed much more complexity, with a much more complex alteration in the NaO_x_/TMO_x_ polyhedron. The electronic structure changes with NaO_x_/TMO_x_ polyhedron variations which lead to rich physics and chemistry for small-molecule adsorption, such as CO_2_ and H_2_O. A more detailed investigation is provided below.

## 6. Electron Density Distribution

The electron density distribution of the (100) surface is calculated from bulk-crystal projection. For the P phase, the Na site showed a slight electron density change. NaMoO_2_ and NaNbO_2_ have the highest and lowest electron densities, respectively. The most striking change in the electron density distribution is from the TMO_6_ octahedron. For the P-phase NaCoO_2_, the calculation showed an elliptical isocycle with a long radius, pointing towards the TM metals. In the P-phase NaNiO_2_, the NiO_6_ octahedron showed less electron localization on the Ni-O bond. The isocycle becomes a truncated cube, which indicates less compression along the c-axis of the crystal. MoO_6_ showed a higher electron density between the oxygen atoms, indicated by the small cycle inside the truncated cube. NbO_6_ showed an even higher electron distribution around the NbO_6_ octahedron, as shown with the darker color around the Nb atom, and the isocycle becomes a rod pointing towards the O atoms, which indicates NbO_6_ compression along the a-b-axis of the crystal. CoO_6_ has the highest electron density for the TM-O bond, while the Ni-O bond has the lowest electron density distribution. The 4d MoO_6_ and NbO_6_ are located at the interval between CoO_6_ and NiO_6_.

**Figure 5:**
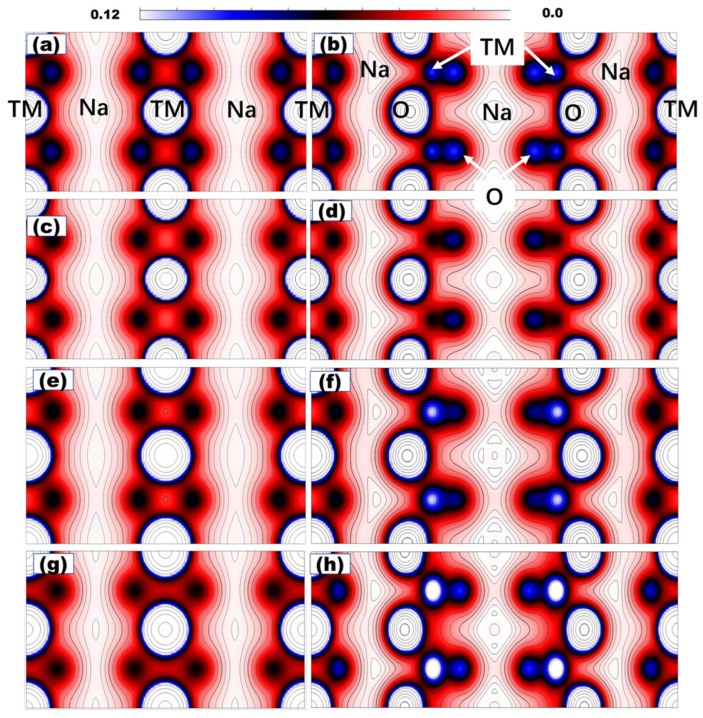
The (100) surface charge density distribution of P- and O-phase (left and right columns, respectively) NaTMO_2_ (TM = Co, Ni, Mo, Nb). (**a**,**b**) for NaCoO_2_; (**c**,**d**) for NaNiO_2_; (**e**,**f**) for NaMoO_2_; (**g**,**h**) for NaNbO_2_. The cycle region is labeled by the elements. The double black–blue cycle is the TM-O bond.

For the O phase, the electron density distributions of Na sites are all different, as shown in the right column of Figure 5. Additionally, the TM-O bond from the double cycle shows a striking difference originating from the different distortions of the TMO_6_ octahedron. In summary, electron density distribution changes with the TM symmetry and element duo to the different TMO_6_ arrangements and TM-O bonding. The difference in the electron density distribution of the (100) surface will lead to rich surface properties that have an important influence on the surface air stability.

The H_2_O/CO_2_ surface interaction comprised chemical bonding and electrostatic and Vdw interactions, which are all strongly affected by the charge density, as shown in Figure 3. The above charge density calculation showed that the charge distribution is entirely different for the NaTMO_2_ crystal (100) surface, even in the Na sites. The charge accumulation patterns on the (100) surface will inevitably affect the interaction [55] between the surface Na/TM site and the H_2_O and CO_2_ through the electrostatic interaction and chemical bonding.

## 7. Projected Density of State (PDOS)

**Figure 6:**
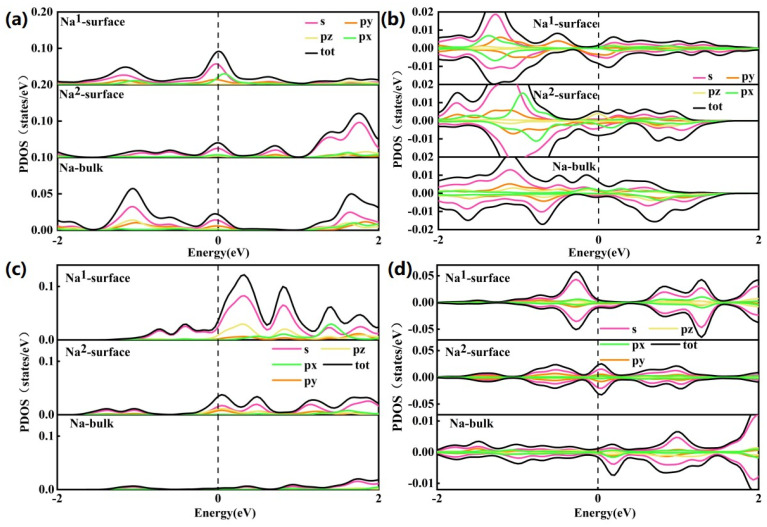
The local projected density of state (PDOS) of surface Na atom with different (100) surfaces: (**a**) NaCoO_2_ (R $\bar{3}$m); (**b**) NaNiO_2_ (R $\bar{3}$m); (**c**) NaNbO_2_ (R $\bar{3}$m); (**d**) NaMoO_2_ (R $\bar{3}$m).

**Figure 7:**
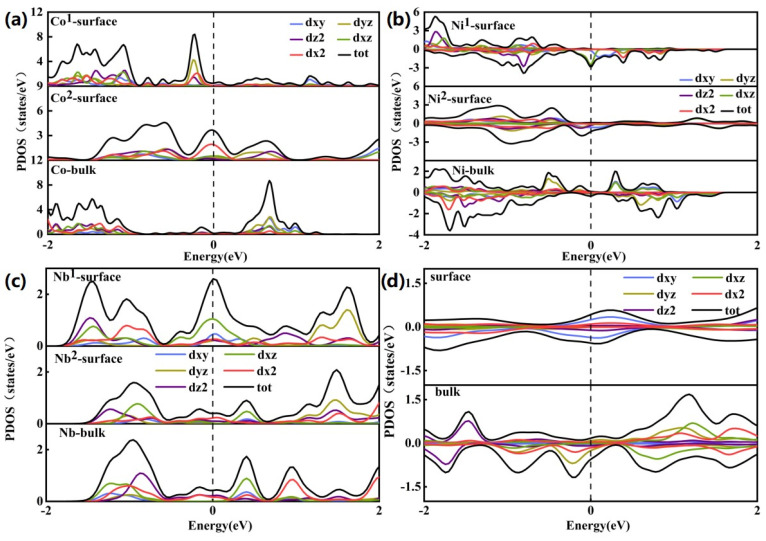
The local projected density of state (PDOS) of surface TM atoms with different (100) surfaces: (**a**) NaCoO_2_ (R $\bar{3}$m); (**b**) NaNiO_2_ (R $\bar{3}$m); (**c**) NaNbO_2_ (R $\bar{3}$m); (**d**) NaMoO_2_ (R $\bar{3}$m).

The above analysis showed that the charge density of the (100) NaTMO_2_ surface has complex patterns with alterations in symmetry, TM element, etc. However, the components of the surface charge density and band center are still unknown, which are important factors for surface adsorption [51]. The projected density of state (PDOS) analysis is employed to provide a detailed overview of the component and band center to understand H_2_O/CO_2_ adsorption. The surface NaCoO_2_ becomes half metallic, as shown in Figure 6 and Figure S14, and our calculations are consistent with previous work [56]. The projected density of state (PDOS) of the various surface Na and TM atoms showed that there are striking PDOS changes between the bulk and surface states (Figure 6 and Figure 7), even for the same Na or TM site at the surface, if they appeared at different surface element terminations, their PDOS are also different (Figure 7 and Figures S14–S22). Therefore, the electron states of surface atoms change with their local environment, such as symmetry, site location, surface termination, etc. For all Na sites, the most prominent phenomenon is that the PDOS increases around the Fermi level compared to bulk Na, which indicates electron accumulation after surface formation. This originated from charge neutralization. The Na ion showed plus charge, and the localization of the electron around the Na ion will decrease the net charge to stabilize the surface. Co showed the same trend as the Na site, while the Ni atom showed more complex behavior. In the O phase, the PDOS around the Fermi level decreased when moving from the bulk to the surface, while in the P phase, the PDOS around the Fermi level increased when moving from the bulk to the surface. Nb showed the same trend as Na, where the PDOS increased around the Fermi level when moving from the bulk to the surface, while the Mo site showed a decreased PDOS around the Fermi level. The calculated PDOS showed there is a unified trend for electron accumulation at the surface atomic site for Na, but the quantity of electron accumulation may be different. The electron distribution for the surface TM sites showed much more complex behavior.

According to the molecular orbital structure, CO_2_ has two occupied nonbonding orbitals from the O oxygen atom as the HOMO level. Such a molecular orbital shape is just like d_yz_/d_xz_ with four lobes and well-separated from the other orbital in energy. H_2_O has only one occupied nonbonding p orbital from the O oxygen atom as the HOMO level with the lobe out of the water molecule plane. However, there is another π orbital just less than 2 eV below the HOMO level [36,37,57], which may involve strong σ-bonding interaction [38,,58]. In most cases, CO_2_ and H_2_O are treated as the Lewis acid and neutral ligand, respectively. According to the metal–ligand interaction, the molecular adsorption energy correlates with the metal orbital energy level and orbital overlap with the empty metal orbital.

**Figure 8:**
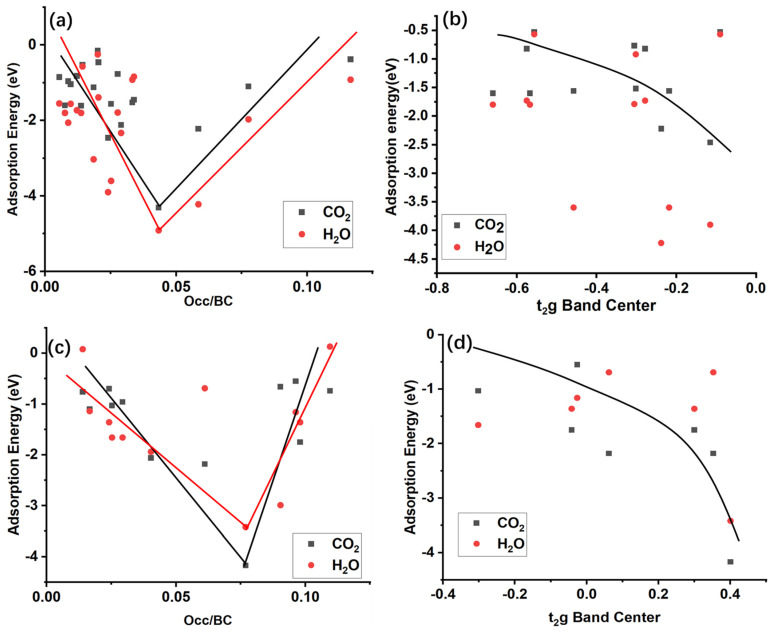
Band occupation and band center dependence of the H_2_O/CO_2_ adsorption energy at different sites of NaTMO_2_. (**a**) CO_2_ and H_2_O adsorption energy of the Na site of NaCoO_2_ and NaNiO_2_. (**b**) CO_2_ and H_2_O adsorption energy of the TM site of NaCoO_2_ and NaNiO_2_. (**c**) CO_2_ and H_2_O adsorption energy of the Na site of NaMoO_2_ and NaNbO_2_. (**d**) CO_2_ and H_2_O adsorption energy of the TM site of NaMoO_2_ and NaNbO_2_. Occ means occupation, and BC indicates the band center of S orbital band.

The orbital center and occupancy of Na s and TM d orbitals around the Fermi level are calculated to assess their correlation with their CO_2_ and H_2_O adsorption energies (Figure 8).

CO_2_ and H_2_O adsorption energies follow the traditional band center theory, which is frequently used in the surface catalysis field. For close comparison of the CO_2_/H_2_O adsorptio energy on the Na site and TM stie between the 3d-NaTMO_2_ (Figure 8a,b) and 4d-NaTMO_2_ (Figure 8c,d) From 3d to 4d element of NaTMO_2_ crystals (100) surface, there is also another notable trend: the relationship between the band center and adsorption energy on Na or TM sites is also shifted to the right. The origin of such a shift is also a very interesting topic for future studies and is currently under investigation. CO_2_ and H_2_O adsorption on the Na site of the (100) surface followed a “V” shape when scaled with the occupation-and-bond-center ratio of the S orbital, which is used to tune the H atom adsorption energy on the MoC_2_ surface [59]. The bottom of the V curve showed very strong adsorption. In such bottom region, water splitting occurs (as shown in Figures S4, S5, S9, S11, and S12), and CO_2_ undergoes multisite adsorption (as shown in Figures S4 and S13). For the adsorption on the TM metal site, CO_2_ showed a monotonically decreasing relationship with an increased t_2_g band center for both the 3d and 4d transition metals. CO_2_ adsorption energy follows the traditional band center theory, which is frequently used in the surface catalysis field [60,61]. However, H_2_O adsorption on the 3d/4d transition metal sites showed a more complex relationship with the band center of d orbitals. Such a relationship and more diverse descriptors need to be explored in more detail, consistent with previous research [62]. The adsorption trend of CO_2_/H_2_O on the Na and TM sites is also different. This originates from the different symmetry of the partially filled Na S orbital and t_2_g orbitals. CO_2_/H_2_O adsorption has totally different orbital overlaps between the Na and TM sites. Another reason is the different electron negativities of the Na and TM elements. When the CO_2_/H_2_O binds with the Na and TM sites, the electron transfer from the site to the CO_2_/H_2_O molecule is different between the Na and TM sites.

## 8. Conclusions

The present study used 3d and 4d sodium layered transition metal oxides to investigate the surface stability of H_2_O and CO_2_ adsorption properties using DFT calculations. This study showed that the material bulk phase (symmetry), surface site, and element type are very important factors for air stability. Contrary to previous experimental results, the P phase is not always more air-stable compared to the O phase, as shown in our calculations, where the O-phase NaNiO_2_ is more stable than the P phase. The electronic structure of the surface atomic site plays a decisive role in the adsorption strength of the CO_2_ and H_2_O molecules. The calculated band center and occupation showed a direct relationship with the adsorption energy. The Na site adsorption for CO_2_ and H_2_O showed the same V-shape trend. However, the TM adsorption for CO_2_ and H_2_O showed a different trend. As the band center of t_2_g increases, the adsorption strength of CO_2_ increases. There is no clear trend for H_2_O adsorption. The band center and occupation near the interval of the Fermi level ([−2, 2] eV in the present study) are strongly correlated with the air stability of the surface. The present study showed that tuning the surface’s electronic structure may be useful for maintaining air stability for the layered transition metal oxide cathode. A more comprehensive study of the air stability of layered transition metal oxides including the other transition metal compoundsis in progress. More importantly, the doping strategy may change the surface electron structure, which can be used to alleviate or prevent air stability issues. The combined ion/electron effect may also contribute to Na ion diffusion, as demonstrated in MnV_2_O_6_ [63]. Thus, how doping affects the Na ion diffusion is another important issue that remains to be explored.

## Data Availability

Data are contained within the article and Supplementary Materials.

## Supplementary Materials

The following supporting information can be downloaded at: https://www.mdpi.com/article/10.3390/nano15141067/s1, Figure S1 Schematic diagram of the P (left) and O (right) phases structures of NaTMO_2_: (a) NaCoO_2_, (b) NaNiO_2_, (c) NaNbO_2_, and NaMoO_2_. Figure S2 surface structure of the P phase NaCoO_2_ and NaNiO_2_ with Na/O and Ni/O termination, respectively. Figure S3 (100) surface energy various NaTMO_2_ (TM = Co, Ni, Mo, Nb) P and O phase. Figure S4 CO_2_ and H_2_O adsorption geometric before and after atomic relaxation (NaCoO_2_-R $\bar{3}$m). Figure S5 CO_2_ and H_2_O adsorption geometric before and after atomic relaxation on the NaCoO_2_ (100) with Co/O atom termination (P63mmc). Figure S6 CO_2_ and H_2_O adsorption geometric before and after atomic relaxation on the NaCoO_2_ (100) with Na/O atom termination (P63mmc). Figure S7 CO_2_ and H_2_O adsorption geometric before and after atomic relaxation (NaNiO_2_-R $\bar{3}$m). Figure S8 CO_2_ and H_2_O adsorption geometric before and after atomic relaxation on the NaNiO_2_ (100) with Na/O atom termination (P63mmc). Figure S9 CO_2_ and H_2_O adsorption geometric before and after atomic relaxation on the NaNiO_2_ (100) with Ni/O atom termination (P63mmc). Figure S10 CO_2_ and H_2_O adsorption geometric before and after atomic relaxation (NaMoO_2_-R $\bar{3}$m). Figure S11 CO_2_ and H_2_O adsorption geometric before and after atomic relaxation (NaMoO_2_-cmcm). Figure S12 CO_2_ and H_2_O adsorption geometric before and after atomic relaxation (NaNbO_2_-R $\bar{3}$m). Figure S13 CO_2_ and H_2_O adsorption geometric before and after atomic relaxation (NaNbO_2_-cmcm). Figure S14 The local projected density of state (PDOS) of surface TM and Na atoms with different (100) surface. (a) NaCoO_2_ (P63mmc-Co/O-termination); (b) NaCoO_2_ (P63mmc-Na/O-termination); (c) NaCoO_2_ (P63mmc-Co/O-termination); (d) NaCoO_2_ (P63mmc-Na/O-termination). Figure S16 The local projected density of state (PDOS) of surface TM and Na atoms with different (100) surface. (a) NaNiO_2_ (P63mmc-Ni/O-termination); (b) NaNiO_2_ (P63mmc-Na/O-termination); (c) NaNiO_2_ (P63mmc-Ni/O-termination); (d) NaNiO_2_ (P63mmc-Na/O-termination). Figure S17 The local projected density of state (PDOS) of surface TM and Na atoms with different (100) surface. (a) NaNbO_2_ (cmcm); (b) NaMoO_2_ (cmcm); (c) NaNbO_2_ (cmcm); (d) NaMoO_2_ (cmcm). Figure S18 The local projected density of state (PDOS) of surface TM and Na atoms with different (100) surface. (a) NaCoO_2_ (R $\bar{3}$m); (b) NaCoO_2_ (P63mmc-Co/O-termination); (c) NaCoO_2_ (R $\bar{3}$m); (d) NaCoO_2_ (P63mmc-Co/O-termination). Figure S19 The local projected density of state (PDOS) of surface TM and Na atoms with different (100) surface. (a) NaCoO_2_ (P63mmc-Na/O-termination); (b) NaNiO_2_ (R3m); (c) NaCoO_2_ (P63mmc-Na/O-termination); (d) NaNiO_2_ (R $\bar{3}$m). Figure S20 The local projected density of state (PDOS) of surface TM and Na atoms with different (100) surface. (a) NaNiO_2_ (P63mmc-Ni/O-termination); (b) NaNiO_2_ (P63mmc-Na/O-termination); (c) NaNiO_2_ (P63mmc-Ni/O-termination); (d) NaNiO_2_ (P63mmc-Na/O-termination). Figure S21 The local projected density of state (PDOS) of surface TM and Na atoms with different (100) surface. (a) NaMoO_2_ (R $\bar{3}$m); (b) NaMoO_2_ (cmcm); (c) NaMoO_2_ (R $\bar{3}$m); (d) NaMoO_2_ (cmcm). Figure S22 The local projected density of state (PDOS) of surface TM and Na atoms with different (100) surface. (a) NaNbO_2_ (R $\bar{3}$m); (b) NaNbO_2_ (cmcm); (c) NaNbO_2_ (R $\bar{3}$m); (d)NaNbO_2_ (cmcm).
