# Supplementary material for: The Air Stability of Sodium Layered Oxide NaTMO2 (100) Surface Investigated via DFT Calculations
Source: Nanomaterials (Basel). 2025 Jul 10;15(14):1067. doi: 10.3390/nano15141067 (PMC12299609; doi:10.3390/nano15141067)
Supplement: Supplementary file 1 [file nanomaterials-15-01067-s001.zip › nanomaterials-3698414-supplementary.pdf]

The air stability of sodium layered oxides $NaTMO_2$ surface investigated by the DFT calculations

Hui Li[1], Qing Xue[1], Shengyi Li[1], Xuechun Wang[2], Yijie Hou[2], Chang Sun[2], Cun Wang[2], Guozheng Sheng[2], Peng Sheng[1], Huitao Bai[1], Li Xu[1*], Yumin Qian[2*]

[1]Beijing Institute of Smart Energy, Beijing, 102209, China

[2]State Grid Smart Grid Research Institute Co., Ltd., Beijing, 102209, China

[3]Key Laboratory of Advanced Optoelectronic Quantum Architecture and Measurement, Ministry of Education, School of Physics, Beijing Institute of Technology, Beijing, 100081 P. R. China

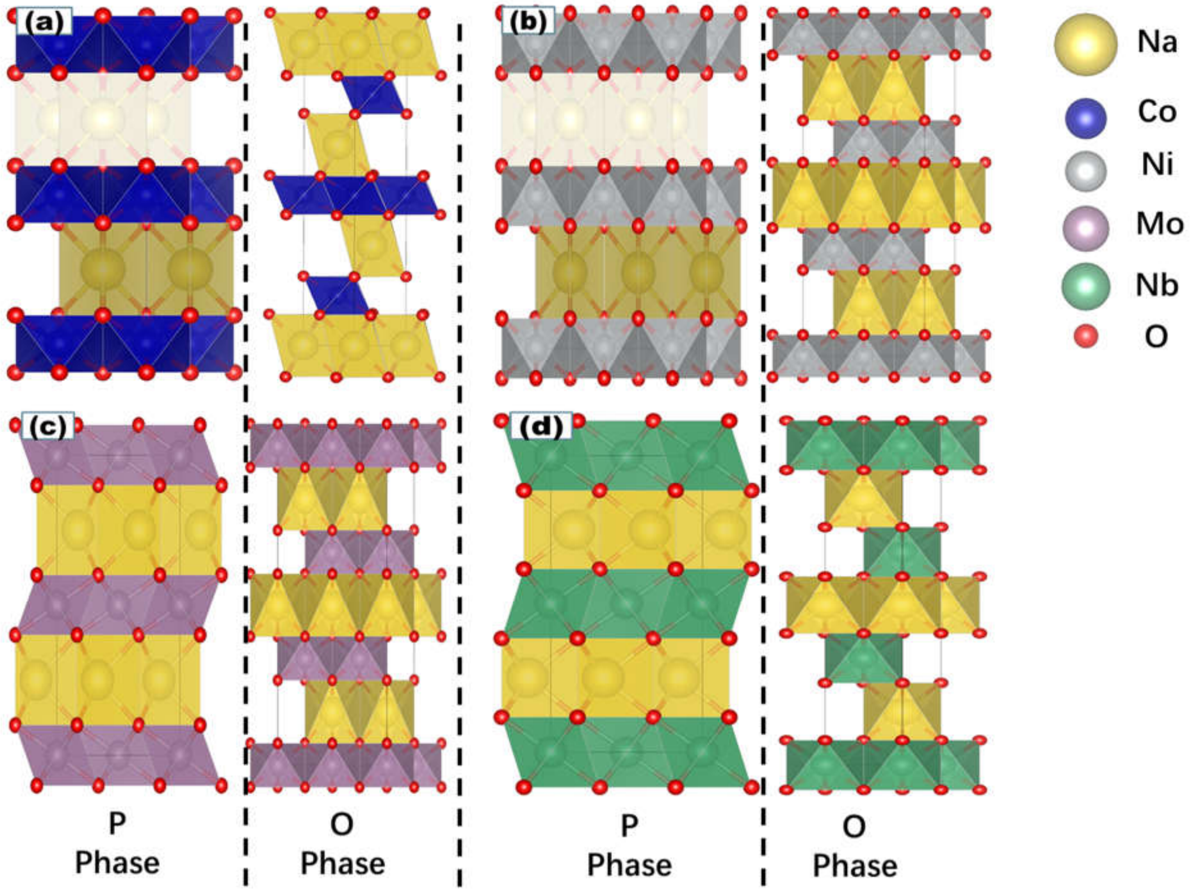

Figure S1 Schematic diagram of the P (left) and O (right) phases structures of $NaTMO_2$: (a) $NaCoO_2$, (b) $NaNiO_2$, (c) $NaNbO_2$, and $NaMoO_2$.

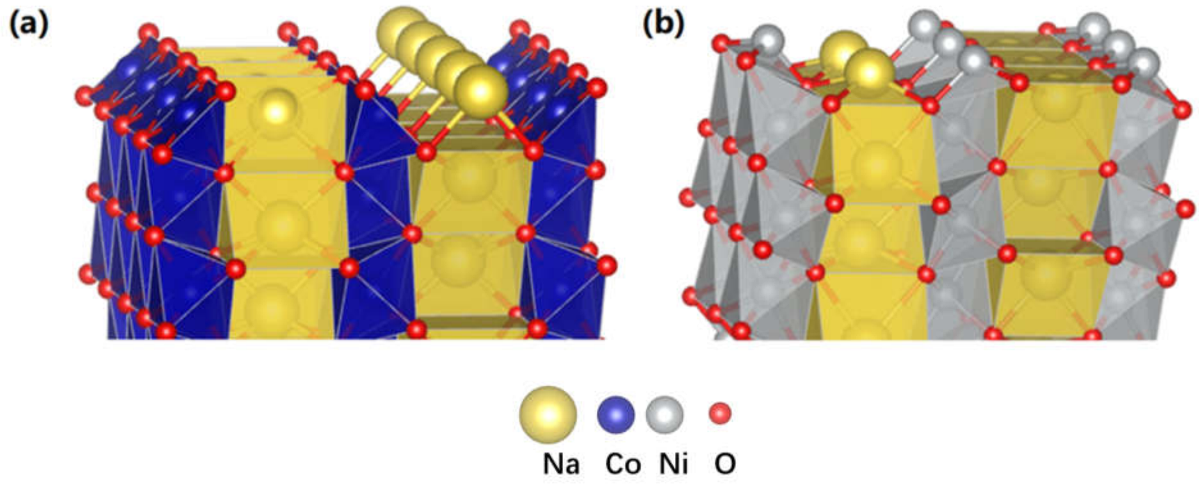

Figure S2 surface structure of the P phase $NaCoO_2$ and $NaNiO_2$ with Na/O and Ni/O termination, respectively.

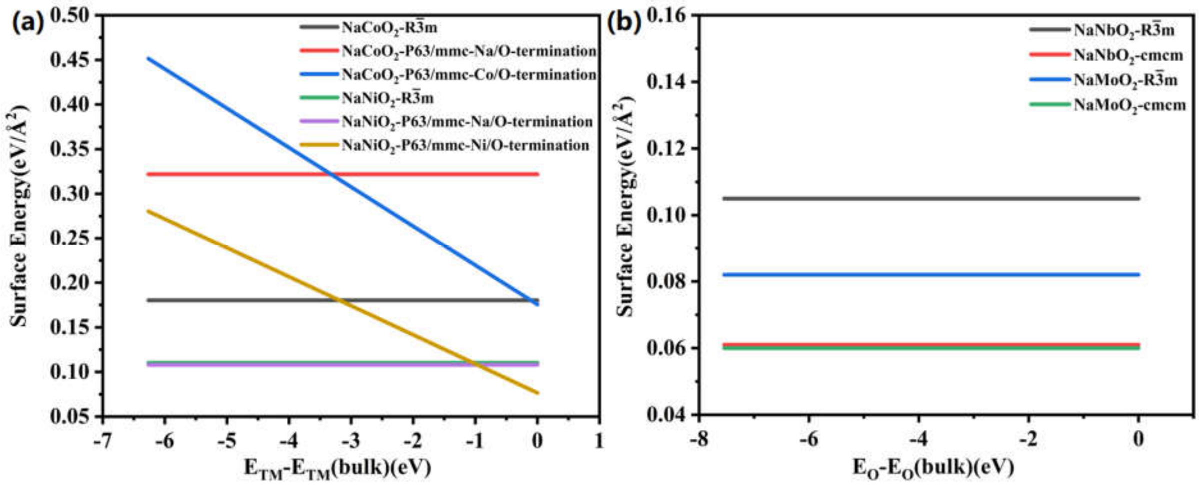

Figure S3 (100) surface energy various $NaTMO_2$ (TM=Co, Ni, Mo, Nb) P and O phase.

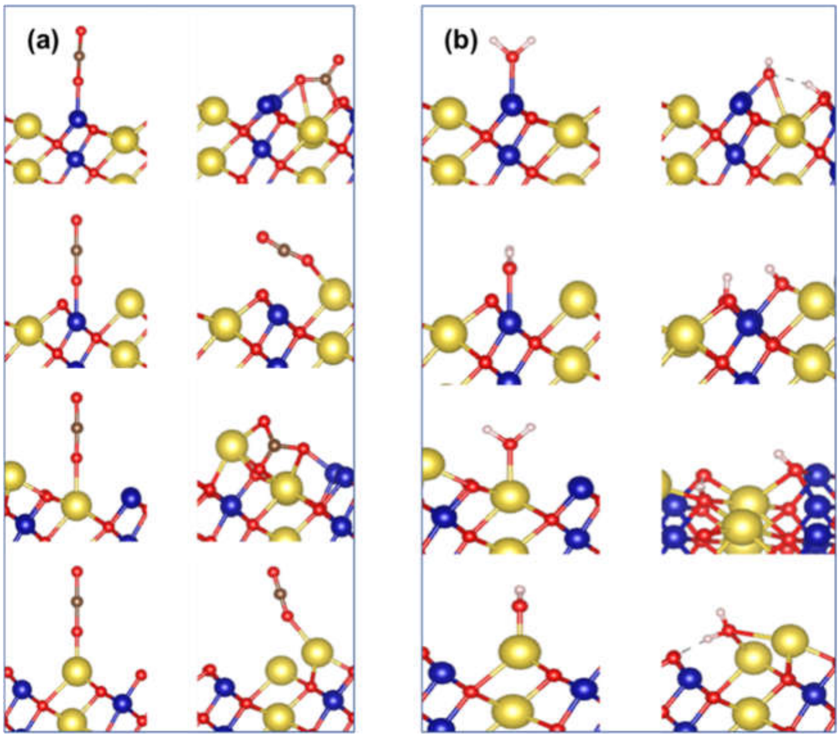

Figure S4 CO_2 and H_2O adsorption geometric before and after atomic relaxation ($NaCoO_2$- $R\overline{3}m$).

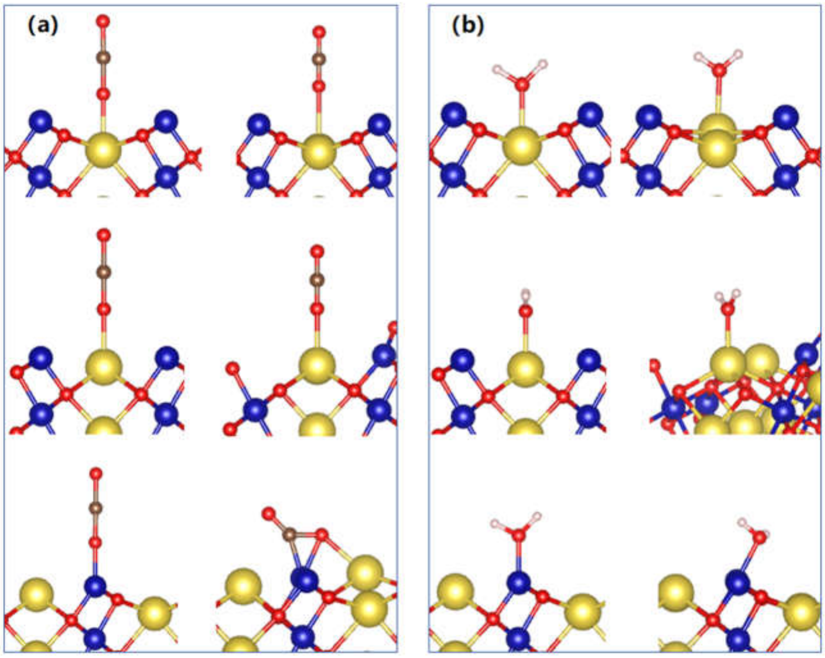

Figure S5 CO_2 and H_2O adsorption geometric before and after atomic relaxation on the $NaCoO_2$ (100) with Co/O atom termination(P63mmc).

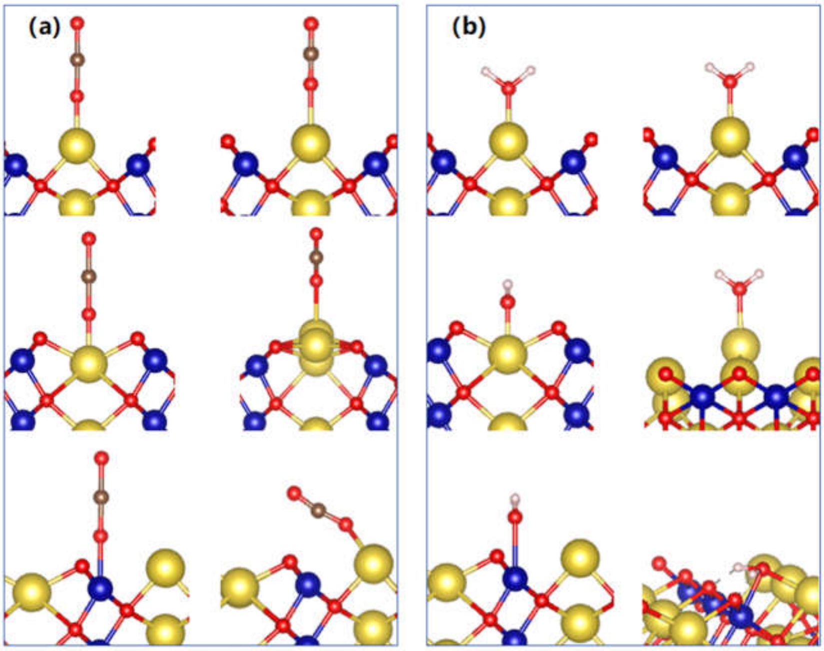

Figure S6 CO_2 and H_2O adsorption geometric before and after atomic relaxation on the $NaCoO_2$ (100) with Na/O atom termination(P63mmc).

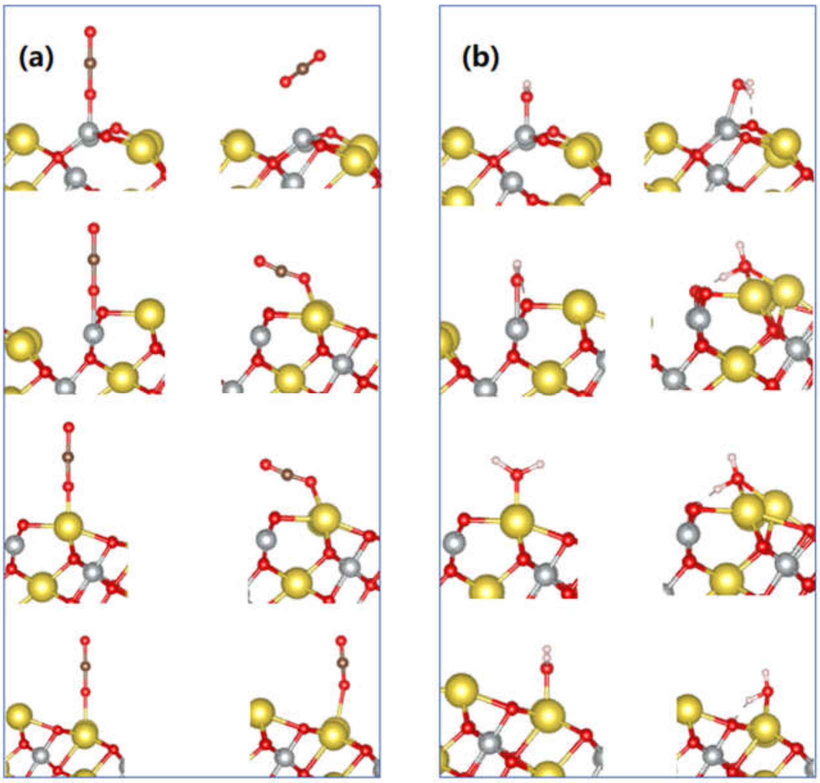

Figure S7 CO_2 and H_2O adsorption geometric before and after atomic relaxation ($NaNiO_2$- R$\overline{3}$m).

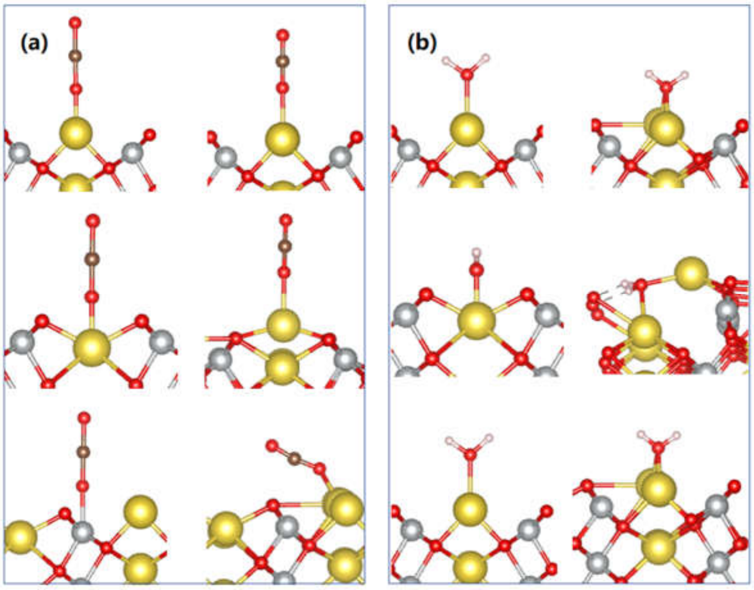

Figure S8 CO_2 and H_2O adsorption geometric before and after atomic relaxation on the $NaNiO_2$ (100) with Na/O atom termination(P63mmc).

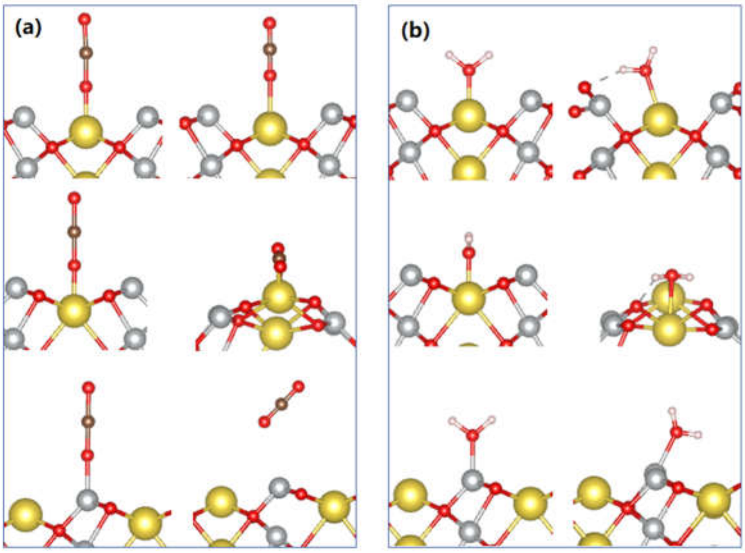

Figure S9 CO_2 and H_2O adsorption geometric before and after atomic relaxation on the $NaNiO_2$ (100) with Ni/O atom termination (P63mmc).

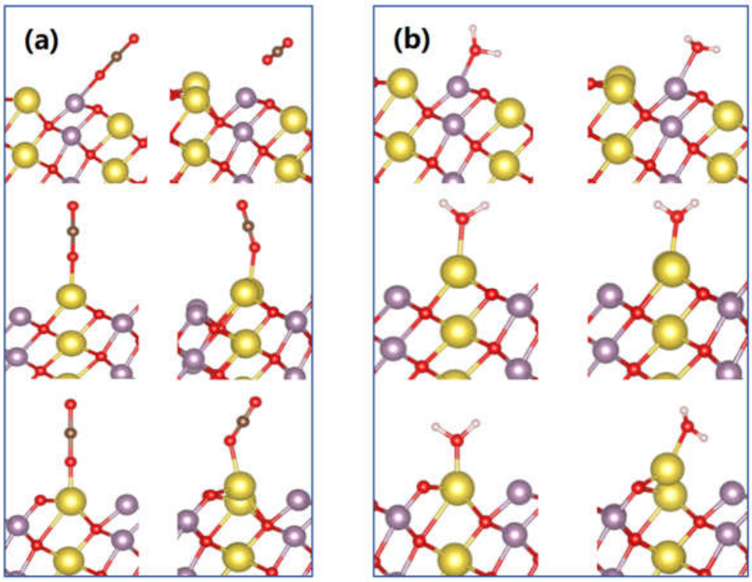

Figure S10 CO_2 and H_2O adsorption geometric before and after atomic relaxation ($NaMoO_2$- $R\overline{3}m$).

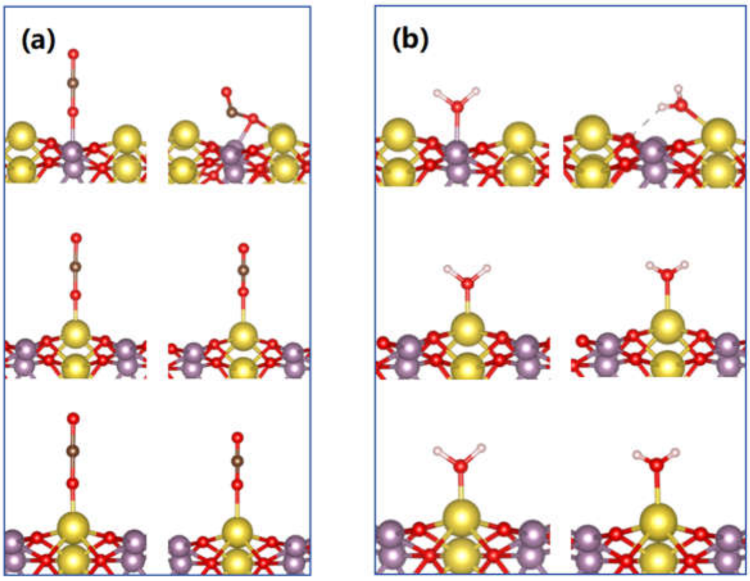

Figure S11 CO_2 and H_2O adsorption geometric before and after atomic relaxation ($NaMoO_2$-cmcm).

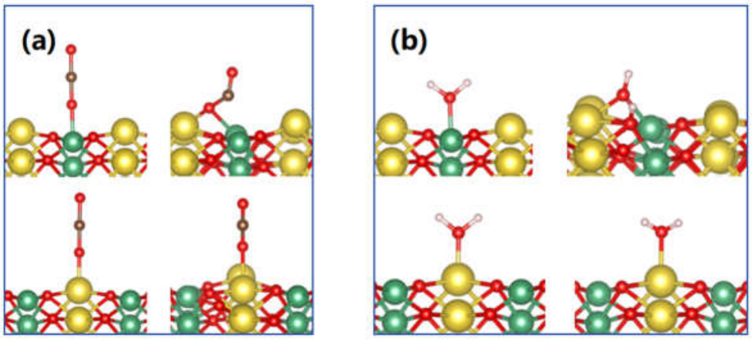

Figure S12 CO_2 and H_2O adsorption geometric before and after atomic relaxation ($NaNbO_2$- R$\bar{3}$m).

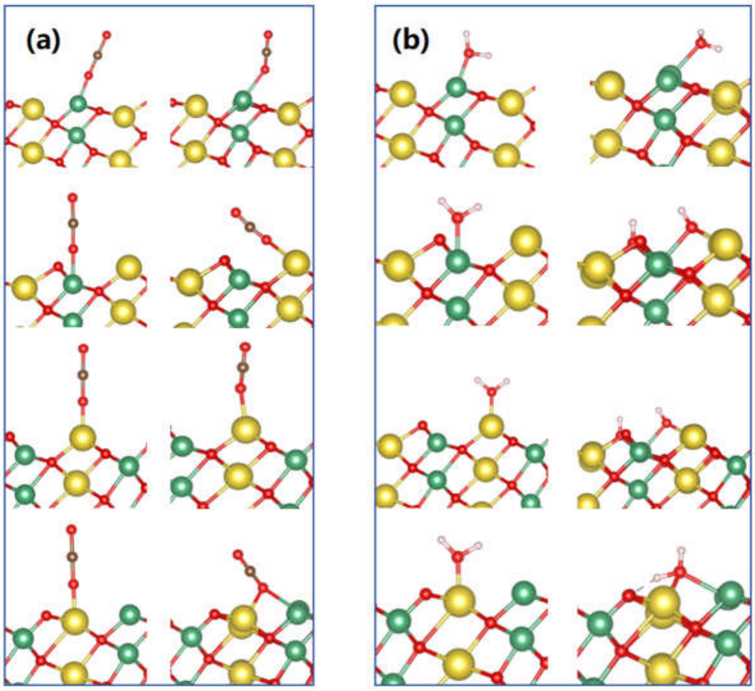

Figure S13 CO_2 and H_2O adsorption geometric before and after atomic relaxation ($NaNbO_2$-cmcm).

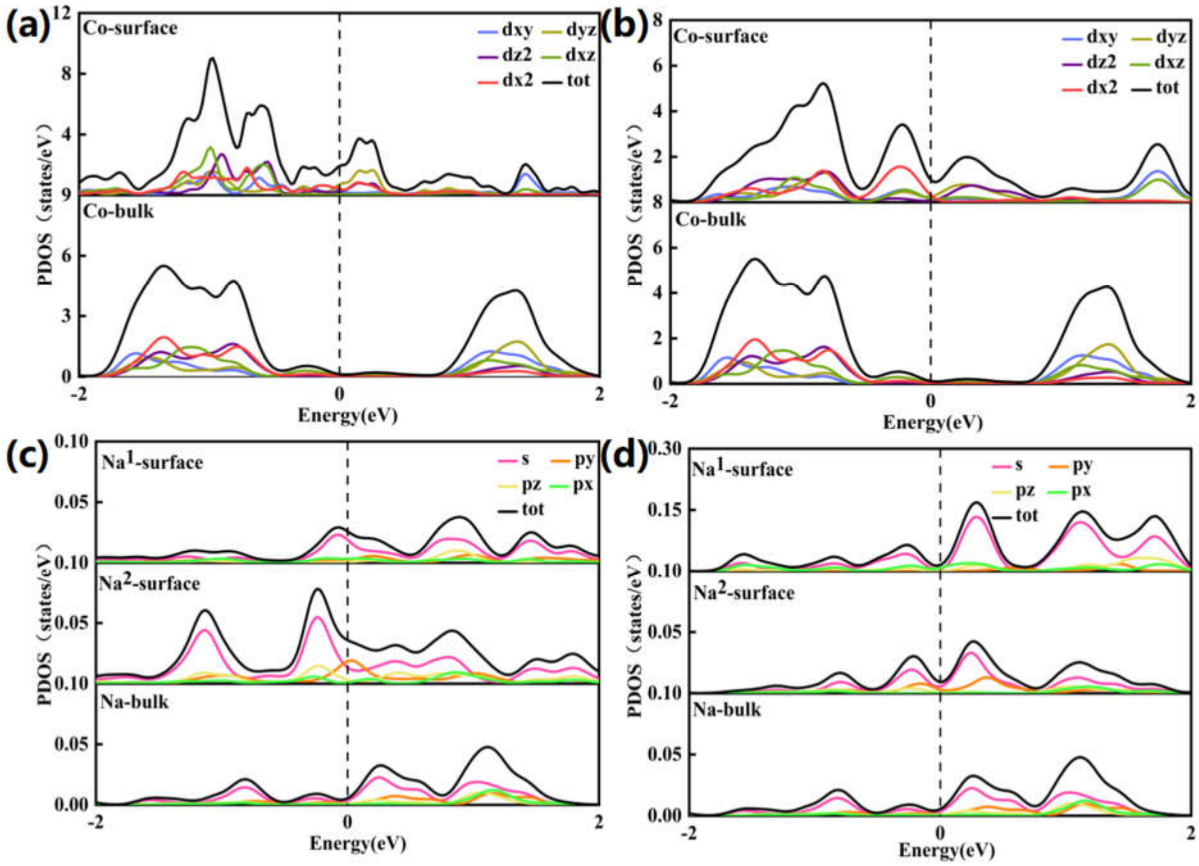

Figure S14 The local projected density of state (PDOS) of surface TM and Na atoms with different (100) surface. (a)$NaCoO_2$(P63mmc-Co/O-termination); (b)$NaCoO_2$(P63mmc-Na/O-termination); (c)$NaCoO_2$(P63mmc-Co/O-termination); (d)$NaCoO_2$(P63mmc-Na/O-termination).

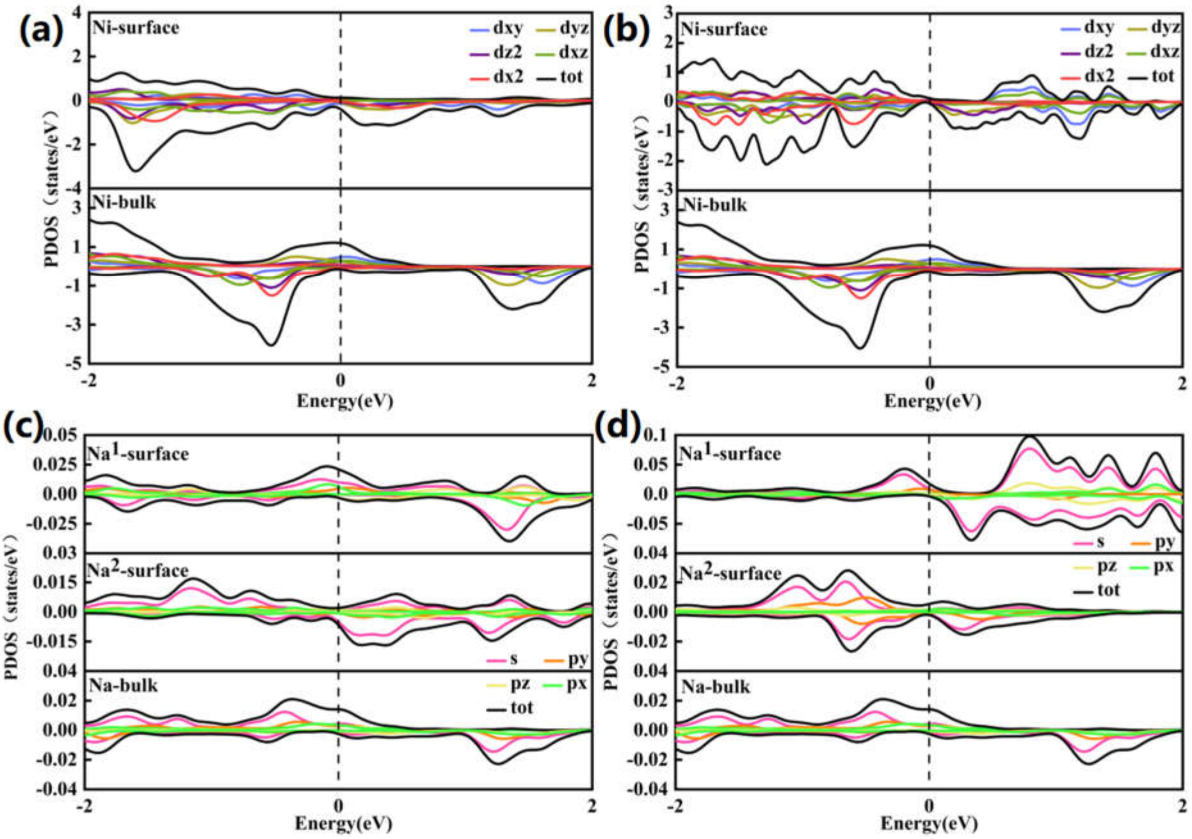

Figure S16 The local projected density of state (PDOS) of surface TM and Na atoms with different (100) surface. (a)$NaNiO_2$(P63mmc-Ni/O-termination); (b)$NaNiO_2$(P63mmc-Na/O-termination); (c)$NaNiO_2$(P63mmc-Ni/O-termination); (d)$NaNiO_2$(P63mmc-Na/O-termination).

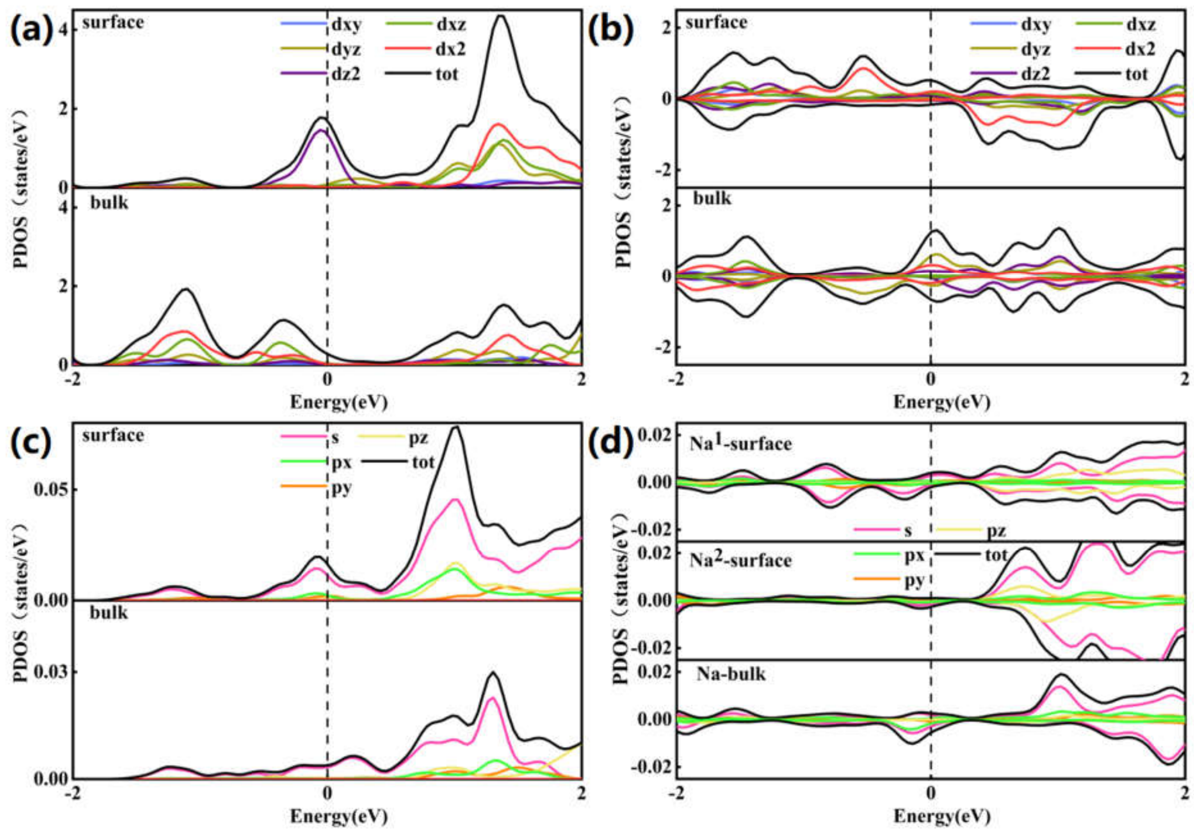

Figure S17 The local projected density of state (PDOS) of surface TM and Na atoms with different (100) surface. (a)$NaNbO_2$(cmcm); (b)$NaMoO_2$(cmcm); (c)$NaNbO_2$(cmcm); (d)$NaMoO_2$(cmcm).

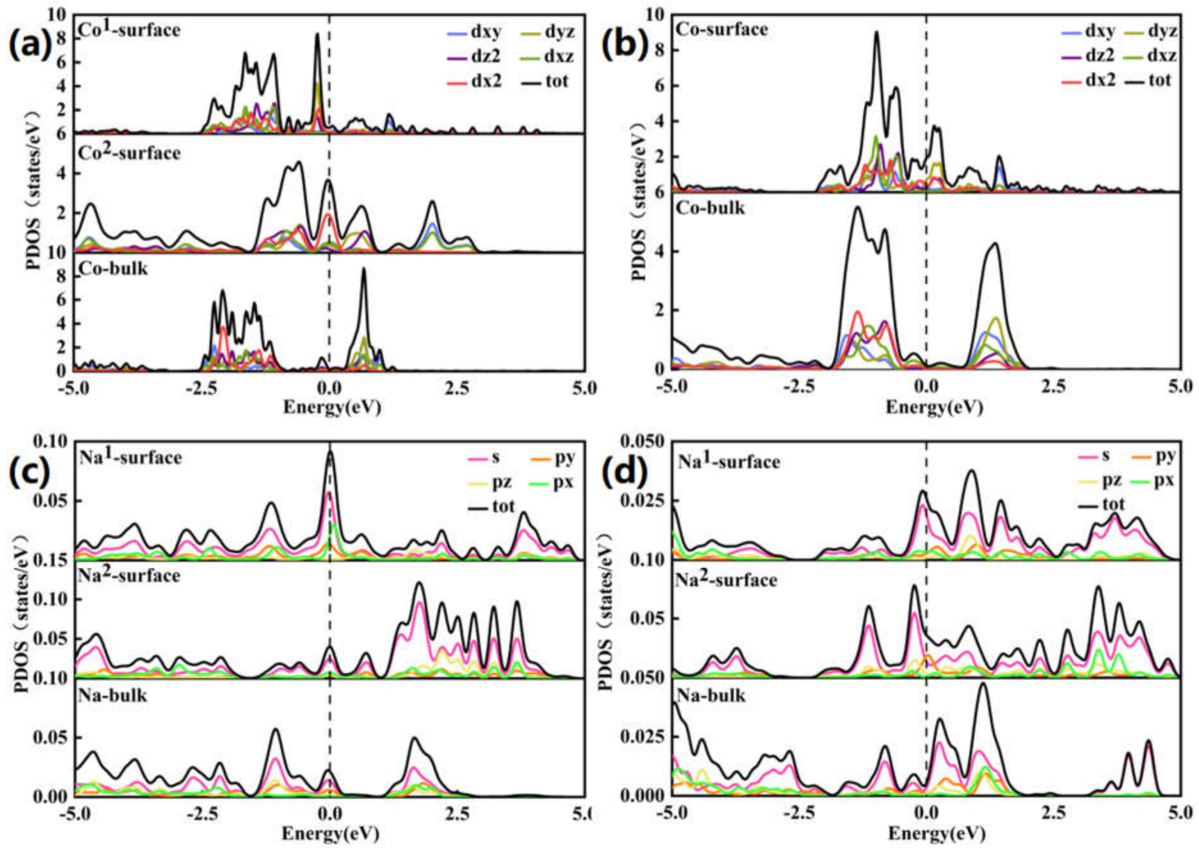

Figure S18 The local projected density of state (PDOS) of surface TM and Na atoms with different (100) surface. (a)$NaCoO_2$(R$\overline{3}$m); (b)$NaCoO_2$(P63mmc-Co/O-termination); (c)$NaCoO_2$(R$\overline{3}$m); (d)$NaCoO_2$(P63mmc-Co/O-termination).

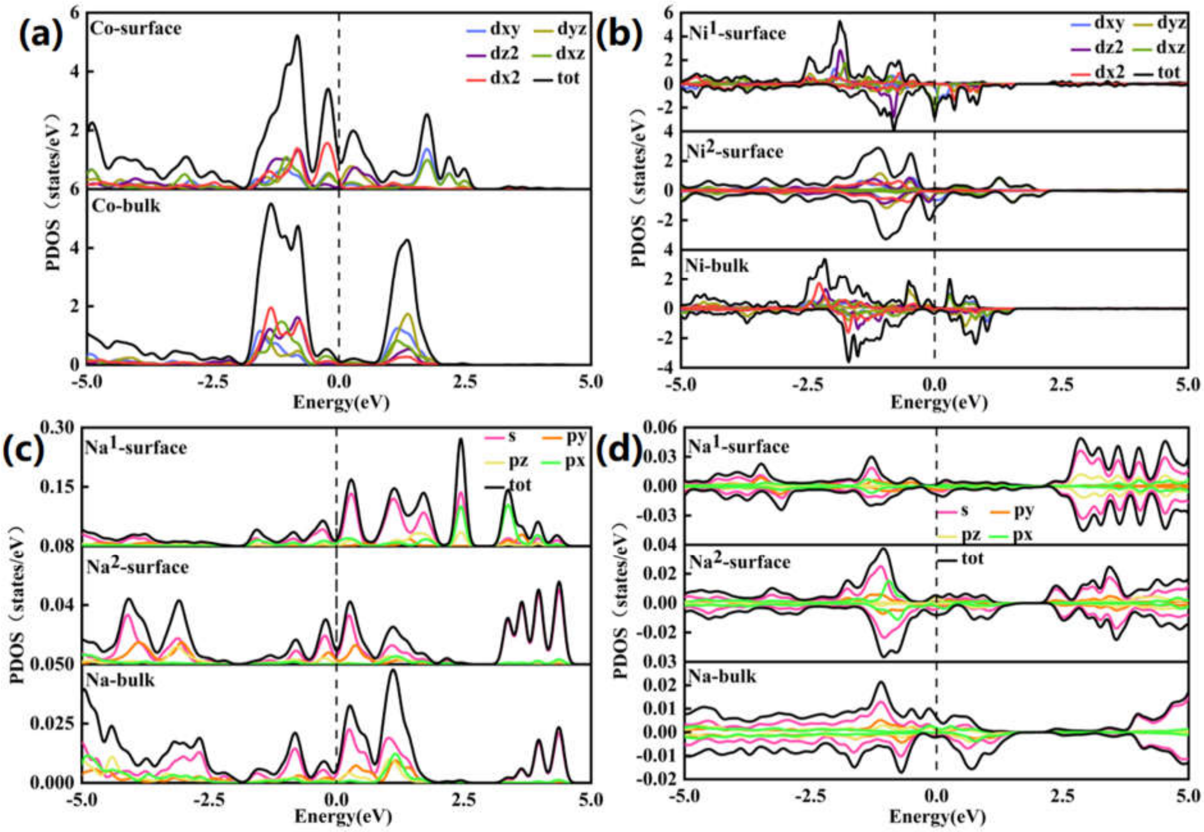

Figure S19 The local projected density of state (PDOS) of surface TM and Na atoms with different (100) surface. (a)$NaCoO_2$(P63mmc-Na/O-termination); (b)$NaNiO_2$(R3m); (c)$NaCoO_2$(P63mmc-Na/O-termination); (d) $NaNiO_2$(R$\bar{3}$m).

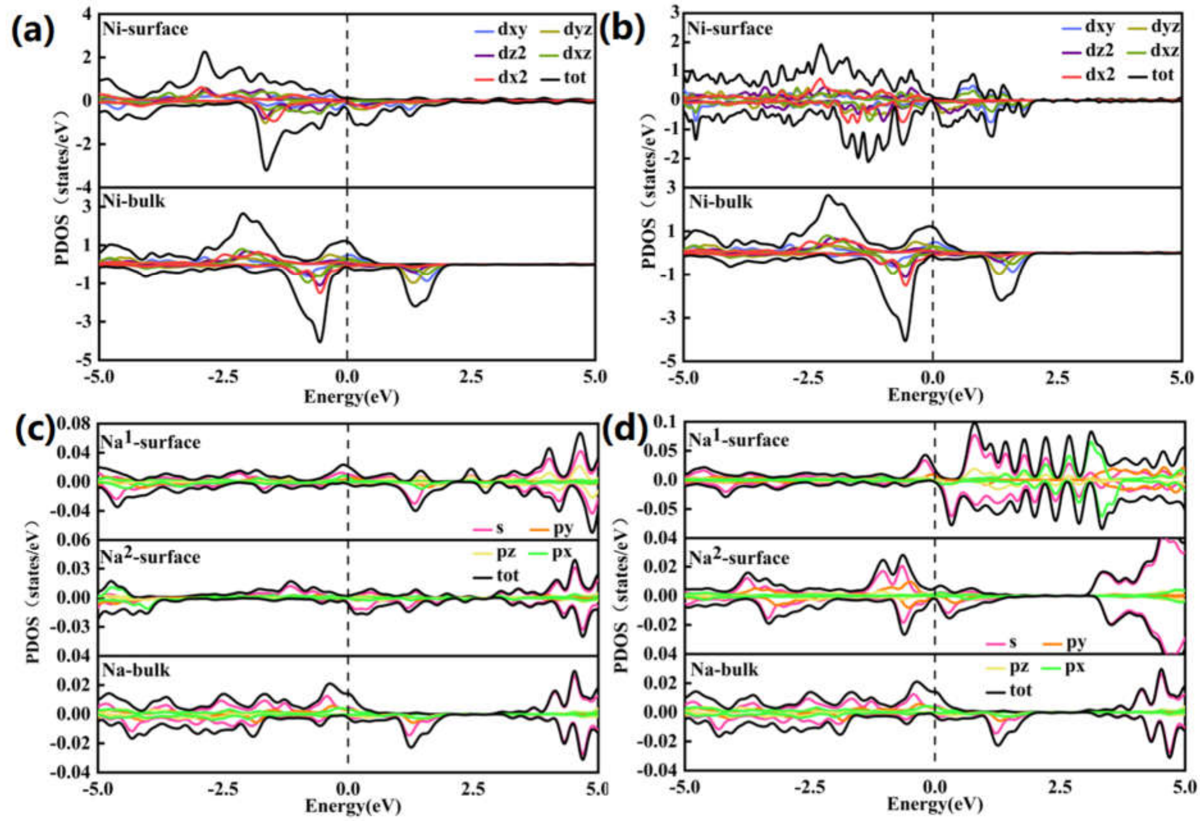

Figure S20 The local projected density of state (PDOS) of surface TM and Na atoms with different (100) surface. (a)$NaNiO_2$(P63mmc-Ni/O-termination); (b)$NaNiO_2$(P63mmc-Na/O-termination); (c)$NaNiO_2$(P63mmc-Ni/O-termination); (d)$NaNiO_2$(P63mmc-Na/O-termination).

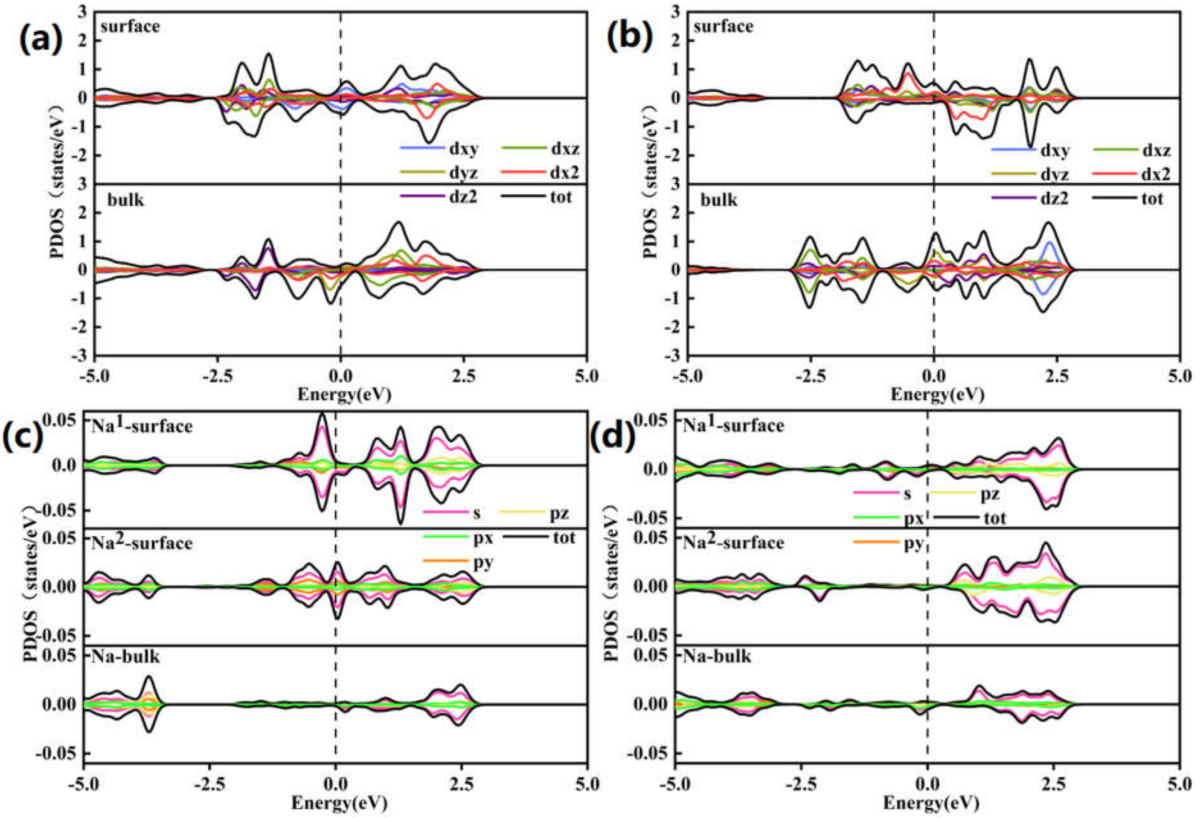

Figure S21 The local projected density of state (PDOS) of surface TM and Na atoms with different (100) surface. (a)$NaMoO_2$($R\bar{3}m$); (b)$NaMoO_2$(cmcm); (c)$NaMoO_2$($R\bar{3}m$); (d)$NaMoO_2$(cmcm).

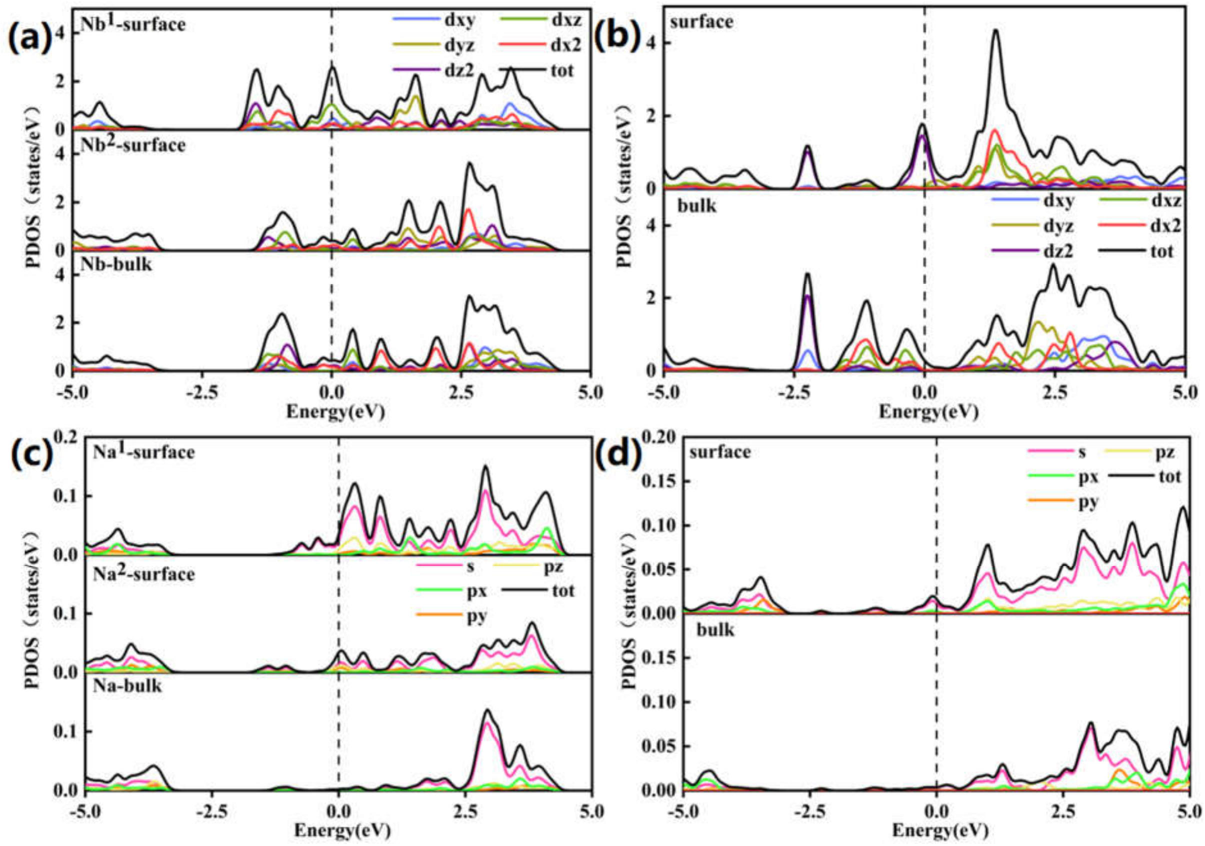

Figure S22 The local projected density of state (PDOS) of surface TM and Na atoms with different (100) surface. (a)$NaNbO_2$(R$\bar{3}$m); (b)$NaNbO_2$(cmcm); (c)$NaNbO_2$(R$\bar{3}$m); (d)$NaNbO_2$(cmcm).
